# Ultrasound-Assisted Water Extraction of Gentiopicroside, Isogentisin, and Polyphenols from Willow Gentian “Dust” Supported by Hydroxypropyl-*β*-Cyclodextrin as Cage Molecules

**DOI:** 10.3390/molecules27217606

**Published:** 2022-11-06

**Authors:** Miloš S. Jovanović, Nemanja Krgović, Katarina Šavikin, Jelena Živković

**Affiliations:** 1 Department of Pharmacy, Faculty of Medicine, University of Niš, Boulevard Dr. Zorana Đinđića 81, 18000 Niš, Serbia; 2 Institute for Medicinal Plants Research “Dr. Josif Pančić”, Tadeuša Košćuška 1, 11000 Belgrade, Serbia

**Keywords:** *Gentiana asclepiadea*, cyclodextrins, UAE, extraction optimization, response surface methodology

## Abstract

The residue after sieving (“dust”) from the willow gentian underground parts is an unexploited herbal tea by-product, although it contains valuable bioactive compounds. Cyclodextrins as efficient green co-solvents, cage molecules, and multifunctional excipients could improve the extraction and contribute to the added value of the resulting extracts. The objective of this study was to determine the optimal conditions for the extraction of gentiopicroside, isogentisin, and total phenolics (TPC) from willow gentian “dust” using ultrasound-assisted water extraction coupled with hydroxypropyl-*β*-cyclodextrin (HP*β*CD). The influence of extraction temperature (X_1_: 20–80 °C), time (X_2_: 20–50 min), and HP*β*CD concentration (X_3_: 2–4% *w*/*v*) was analyzed employing the response surface methodology (RSM). The optimal extraction conditions for simultaneously maximizing the extraction yield of all monitored responses were X_1_: 74.89 °C, X_2_: 32.57 min, and X_3_: 3.01% *w*/*v*. The experimentally obtained response values under these conditions (46.96 mg/g DW for gentiopicroside, 0.51 mg/g DW for isogentisin, and 12.99 mg GAE/g DW for TPC) were in close agreement with those predicted, thus confirming the suitability and good predictive accuracy of the developed RSM models. Overall, the developed extraction system could be an applicable alternative strategy to improve the extraction of bioactive compounds from the underutilized “dust” of willow gentian underground parts.

## 1. Introduction

*Gentiana asclepiadea* L. (Gentianaceae), traditionally known as willow gentian, grows in the mountains of Southern and Central Europe [1]. Its underground parts, i.e., rhizomes and roots, are traditionally used in the form of aqueous infusion or water-ethanolic macerate as a bitter tonic and for the treatment of different gastrointestinal disorders, especially liver diseases [2,3]. Moreover, due to a bitterness, it is also used in the beer industry or for food flavoring [4].

Willow gentian underground parts are characterized by a similar phytochemical profile to officinal *Gentiana lutea*. The main classes of bioactive compounds are secoiridoids, xanthones, and flavonoid *C*-glycosides [1]. Secoiridoid glycosides such as gentiopicroside, sweroside, swertiamarin, and amarogentin, widespread among *Gentiana* species, are responsible for the bitter taste and high range of biological activities, while xanthones such as isogentisin are chemotaxonomically and pharmacologically valuable metabolites [5]. There are reports about hepatoprotective [6], antigenotoxic [4], antihyperglycemic [7], anti-noroviral [8], anthelmintic, antimicrobial, and antioxidant activity [9,10,11].

Besides the comprehensive research in the field of internal medicine branches, there is increased interest in *Gentiana* species for possible application in skin disorders and healing wounds inspired by ethnomedical data [12]. Recently, it has been proven that gentiopicroside, the main secondary metabolites of *G. asclepiadea* underground parts, accelerates cutaneous wound healing by stimulating re-epithelialization, the development of granulation tissue, and enhanced collagen synthesis [13]. Gentiopicroside has also been reported to exhibit antipruritus, antipain, and reparative effects in corticosteroid addictive dermatitis [14]. Modern molecular modelling methods predicted that natural secoiridoids could be potent inhibitors of matrix metalloproteinases (the most potent among them were amarogentin and gentiopicroside), indicating their skin anti-photoaging potential [15]. *G. lutea* extract finds application in atopic eczema and very dry skin treatment [16], while *G. scabra* underground parts have a potential as an anti-inflammatory agent in contact dermatitis [17]. Overall, skin protection appears to be one of the new promising chapters in *Gentiana* phytotherapy.

The conventional extraction methods are accompanied by disadvantages such as time, energy, and organic solvent consumption and limited applicability of resulting extracts in pharmaceutical, cosmetic, and nutritional formulations. In the context of environmental and sustainability concerns, time-saving ultrasound-assisted extraction (UAE) has been established as an innovative extraction technique that is usable on an industrial scale [18]. The UAE is particularly appropriate for coarse raw materials such as roots and rhizomes, because it provokes detexturation of the plant matrix by acoustic cavitation accelerating the extraction of bioactive compounds [19]. The use of pure water as a biocompatible, non-flammable, non-corrosive, cheap, and widely available solvent is absolutely in line with green principles. However, water is a poor solvent for the extraction of less-polar compounds. These shortcomings can be solved by using co-solvents [20].

The cutting-edge approach to enhancing the solubility of phenolics in water is hiring cyclodextrins (CDs) as co-solvents and cage molecules, mainly *β*-cyclodextrin (*β*CD) and hydroxypropyl-*β*-cyclodextrin (HP*β*CD). CDs are cyclic oligosaccharides with an external hydrophilic surface and an internal less-hydrophilic cavity, which are capable of forming inclusion complexes with guest molecules, providing better water solubility [21]. Beyond solubility enhancement, CDs may protect molecules against harmful environmental impacts (light, temperature, pH, and moisture-induced degradation), thereby extending the shelf life of products. CDs can modify physicochemical and biological properties, mask off-odor and taste, improve bioavailability, and modulate the delivery rate of bioactive compounds from drug/food formulations [22]. Considering the growing interest for *Gentiana* species in cosmetology, it should be noted that CDs can be used to prepare multiple emulsions in the absence of classic surfactants [23]. Additionally, water-CDs extracts would be preferred over most commonly used water-ethanol extracts, bearing in mind the undesirability of ethanol in cosmetic formulations and the negative influence of ethanol itself on wound healing. Thus, the water-CDs extracts would be directly applicable in cosmetic formulations and thus allow bypassing the difficulties of bioactive compounds recovery. Furthermore, CDs can enhance the topical delivery of drugs [24]. Moreover, our previous study confirmed that microencapsulation by spray-drying is an effective approach to preserve the bioactive compounds of willow gentian, and CDs could be considered as novel carriers for such systems that can overcome the conventional ones [25]. Overall, CDs can be considered multipurpose excipients.

Taking into account that the phytochemical profile of extracts depends on numerous parameters, such as power and frequency of the ultrasound, the type of solvent, solid-to-liquid ratio, extraction time, particle size, temperature, and presence of co-solvents extraction, optimization of operating conditions is a rational approach to provide a highly efficient process [18,26]. The traditional “one-factor-at-a-time” optimization approach is time- and material-consuming and inefficient for evaluating the interaction effect of process parameters. These limitations can be overcome by employing statistical and mathematical tools such as the response surface methodology (RSM) [27]. The Box–Behnken design (BBD) and central composite design (CCD) are the most commonly used response surface designs for the optimization of the extraction process. Compared to CCD, BBD is more economical since it requires a smaller number of experimental processes [18]. Another advantage of the BBD is that it does not contain combinations for which all factors are simultaneously at their highest or lowest levels. So, these designs are useful in avoiding experiments performed under extreme conditions, for which unsatisfactory results might occur [28].

Due to the increasing production of herbal teas, consequently, herbal “dust” after sieving remains. Although it is treated as a waste and economic and environmental inevitability, it could find value-added applications. New trends considering industrial ecology concepts have pointed out the need to recognize waste as a source of biologically valuable compounds and to recover them [29].

The current study aimed to optimize the HP*β*CD-aided water UAE process for recovery of the main secoiridoid gentiopicroside, xanthone isogentisin, and total polyphenols (TPC) from willow gentian underground parts “dust” (particles smaller than 0.75 mm). The impacts of extraction time, temperature and the concentration of HP*β*CD as independent variables on the target compounds yields (response variables) were investigated using RSM. To the best of our knowledge, there is no information on the application of HP*β*CD as a booster of UAE for water extraction of bioactive compounds from any *Gentiana* species and especially of the remains (“dust”) after primary processing for teas production.

## 2. Results and Discussion

### 2.1. Model Assessment

The interest in the investigation of polyphenolic compounds is based on the knowledge of their protective effects at the biological level. Therefore, maximizing their extraction efficiency is the key to providing therapeutic effects. Conventional extraction techniques might result in degradation of the bioactive molecules, thereby reducing their bioavailability. CDs are well known for their ability to build inclusion complexes with bioactive compounds, thus improving their solubility, stability, and bioavailability. According to Gao et al. [30], UAE is pointed out as a significant technique for enhancement of the extraction efficacy of bioactive components using an aqueous CD solution as an extraction solvent. Recent studies on HP*β*CD-aided polyphenol extraction demonstrated the importance of its concentration on the extraction performance [31,32]. Moreover, the ultrasonication time, as well as temperature, are parameters whose effects on the extraction efficiency are undoubted, and they were nominated as the independent variables in our study. The values of these variables used in the RSM in order to optimize the TPC, gentiopicroside, and isogentisin content of the willow gentian extract are shown in Table 1.

The effects of each of the mentioned extraction factors on monitored responses were analyzed using ANOVA, and the significance of each coefficient was determined using *p*-values. Normal probability plots for all models are presented (Figures S1–S3, Supplementary Materials). As reported by ANOVA analysis (Table 2), the experimental model was significant since the *p*-values of the models were less than 0.05 at a 95% confidence level (<0.0001, 0.0008 and 0.0004 for gentiopicroside, isogentisin and TPC, respectively). Relatively high values of coefficient of determination (R^2^) and adjusted R^2^ for all responses indicate that the experimentally obtained values fitted well with the generated models. However, slightly lower values of predicted R^2^ imply that the model’s ability to predict the new experimental points could be compromised. Therefore, the generated empirical models should be experimentally confirmed, which was done (Section 2.3). As shown in Table 2, the lack-of-fit values were non-significant (for the models for gentiopicroside, isogentisin and TPC, they were 0.1436, 0.3397, and 0.1760, respectively), showing the best fitness of the model and its adequacy to project the outcomes.

### 2.2. Influence Analysis

#### 2.2.1. Effect of Extraction Parameters on Total Phenolics Content

The extraction yield of TPC from the underground parts of willow gentian ranged from 10.55 to 13.18 mg GAE/g DW. The results obtained were slightly higher compared to those obtained by UAE using water-ethanol as a solvent [26]. According to them, the extraction yield of TPC from the underground parts of the willow gentian varied from 5.64 to 10.26 mg GAE/g DW. In the current study, the highest TPC was obtained using 20 min of ultrasonication at 80 °C and a HP*β*CD concentration of 3% (*w*/*v*). On the contrary, the lowest TPC was obtained by applying an extraction time of 35 min at 20 °C and a HP*β*CD concentration of 4% (*w*/*v*).

The significance of each independent variable in the model can be evaluated based on their *p*-values obtained using ANOVA analysis (if *p* < 0.05, the term will have a remarkable effect). From the ANOVA results, it was evident that linear term X_1_, quadratic terms X_1_^2^ and X_3_^2^, and interaction term X_1_X_2_ significantly affected TPC extraction efficiency. The final predictive equation that describes the influence of process parameters on the extraction efficiency of TPC from the willow gentian underground parts is as follows:

TPC (mg GAE/g DW) = 12.55 + 0.63X_1_ + 0.09X_2_ – 0.22X_3_ – 0.59X_1_X_2_ – 0.40X_1_^2^ – 0.38X_3_^2^ (1)

**Figure 1:**
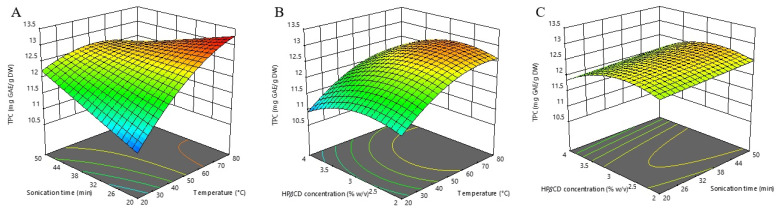
Three-dimensional response surfaces plots of total phenolics (TPC) as a function of sonication time/temperature (**A**); hydroxypropyl-β-cyclodextrin (HPβCD) concentration/temperature (**B**); and HPβCD concentration/sonication time (**C**).

In Figure 1, we have demonstrated the influence of investigated extraction parameters on TPC. It is evident that the most dominant factor influencing TPC extraction was extraction temperature, and this factor affected TPC positively. This is consistent with the findings of the aforementioned study [26], where the temperature was also the most influential factor affecting TPC extraction. High extraction temperatures enhance the solubility and diffusion of polyphenols [33]. An increased temperature improved the efficiency of polyphenolics extraction through enlarging the surface contact area and reducing the viscosity and density of the applied solvent. That way, the established conditions promoted the release of bounded phenolics from plant material and damaged the cell wall integrity, consequently intensifying solubility and coefficients of diffusion [34]. However, the negative quadratic effect of extraction temperature suggests that excessive heating leads to the opposite effect. This is in agreement with the report of Arruda et al. [35], that a temperature above 75 °C can cause degradation of polyphenols and/or enhance their interaction with other matrix compounds, leading to slower extraction. In addition, the observed negative interaction between temperature and time (X_1_X_2_) indicates that the positive influence of temperature decreases when the extraction time is at its higher level, and vice versa. From this perspective, extraction at higher temperatures requires a shorter time. Another noteworthy finding, the statistically significant X_3_^2^ term in the negative mode indicates that excessive concentration of HP*β*CD leads to lower TPC extraction yield. Although contrary to expectations, this effect could be partially explained by the competition between HP*β*CD and phenolic compounds during dissolving in the water solvent. Such a complex influence analysis, which is not possible if the optimization is carried out with the classical “one-factor-at-a-time” approach, fully highlights the advantages of the RSM.

According to our results, the calculated optimal conditions for maximizing the individual TPC response are an extraction temperature of 80 °C, an extraction time of 20 min, and a HP*β*CD concentration of 3.00% (*w*/*v*), with the predicted value of 13.275 mg GAE/g DW.

#### 2.2.2. Effect of Extraction Parameters on Gentiopicroside Content

The most abundant secoiridoid compound in willow gentain underground parts is gentiopicroside, and the highest extraction yield of this compound (49.03 mg/g DW) was noted at an extraction time of 20 min, an extraction temperature of 80 °C and a HP*β*CD concentration of 3% (*w*/*v*). On the contrary, the lowest gentiopicroside content (35.74 mg/g DW) was obtained under the following experimental conditions: extraction time of 35 min, extraction temperature of 20 °C and HP*β*CD concentration of 4% (*w*/*v*). Recorded values were in accordance with our previous study, where content of this compound in willow gentain underground part extracts obtained using the UAE ranged from 32.41 to 50.75 mg/g DW [26]. Similarly, Olennikov et al. [7] achieved gentiopicroside content of 64.71 mg/g DW using UAE with 60% methanol, an extraction temperature of 40 °C, a solid-to-solvent ratio of 1:20 (*w*/*v*), and an extraction time of 60 min.

**Figure 2:**
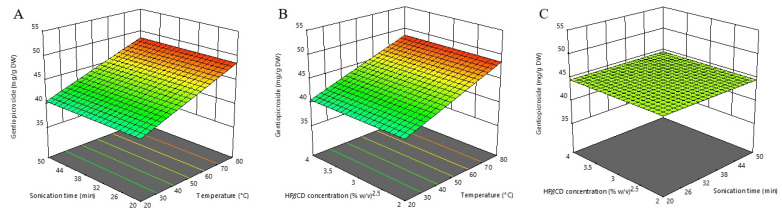
Three-dimensional response surfaces plots of gentiopicroside as a function of sonication time/temperature (**A**); hydroxypropyl-β-cyclodextrin (HPβCD) concentration/temperature (**B**); and HPβCD concentration/sonication time (**C**).

According to the ANOVA results in our study, it was evident that a significant positive impact on gentiopicroside content was exerted only by the linear term of the extraction temperature. This is also evident from Figure 2, which was prepared in order to visualize the relationship between gentiopicroside content and selected process variables. On the other hand, the extraction time, the HP*β*CD concentration, and the interaction between tested variables showed no significant effects in the estimated experimental range. The developed predictive equation for gentiopicroside extraction is stated below:

Gentiopicroside (mg/g DW) = 44.59 + 3.99X_1_ (2)

The insignificant effect of HP*β*CD concentration can be attributed to the good water solubility of gentiopicroside itself, thus making dissolution support redundant. The prominent effect of extraction temperature is consistent with previous studies in which UAE optimization of *G. lutea* root was performed [27,36], where the maximum amount of gentiopicroside content was obtained using the highest tested temperatures (65 and 80 °C, respectively). Also, Ariño et al. [37] demonstrated that the content of this secoiridoid compound in yellow gentian root extracts continuously rose with the increase of temperature up to 75 °C. Contrary to our result, Dang et al. [38] reported a significant effect of extraction time on the extraction efficiency of gentiopicroside. Prolonging sonication improved the extraction of gentiopicroside from *G. straminea*. The peak value was reached at about 75 min and did not change significantly after that time. The reason for the statistically insignificant effect of extraction time reported in our study could be found in the fact that we used “dust” powdered plant material. Since this plant matrix was significantly destructured, a large amount of gentiopicroside could rapidly pass into the extract during the short-term “washing phase” and quickly reach a peak value that was followed by a “plateau phase”. Our experimental range (20–50 min) probably covered the mentioned plateau phase of the extraction where saturation was reached, and therefore there were no significant changes in the gentiopicroside content over time.

Our study demonstrated that the optimal conditions for maximizing the individual gentiopicroside response are an extraction temperature of 80 °C, an extraction time of 50 min, and a HP*β*CD concentration of 3.00% *w*/*v*, with the predicted response of 48.578 mg/g DW.

#### 2.2.3. Effect of Extraction Parameters on Isogentisin Content

The extraction efficiency of isogentisin obtained from the willow gentian underground part utilizing HP*β*CD coupled with UAE ranged from 0.37 to 0.57 mg/g DW. The maximal isogentisin content was achieved by application of the extraction temperature of 50 °C, extraction time of 35 min, and HP*β*CD concentration of 3% (*w*/*v*). The minimal isogentisin content was obtained after an application extraction temperature of 20 °C, an extraction time of 35 min, and a HP*β*CD concentration of 2% (*w*/*v*). In our earlier works, mainly higher contents of isogentisin were obtained from the underground parts of *G. lutea* and *G. asclepiadea*, but after using water-ethanol as the solvent [26,27]. On the other hand, the conditions that we defined in this work for the maximal water extraction of isogentisin from willow gentian “dust” allowed for a larger amount of this compound than after using 10% of ethanol (minimum tested concentration) in our previous study on willow gentian underground parts (0.46 mg/g DW) [27].

According to ANOVA, the most influential factor was extraction temperature (linear and quadratic terms), followed by HP*β*CD concentration (quadratic term) and extraction time (linear term). The developed model equation describing the relationship between isogentisin extraction efficiency and monitored extraction conditions is shown below:

Isogentisin content (mg/g DW) = 0.53 + 0.04X_1_ + 0.02X_2_ + 0.01X_3_ – 0.06X_1_^2^ – 0.03X_3_^2^ (3)

**Figure 3:**
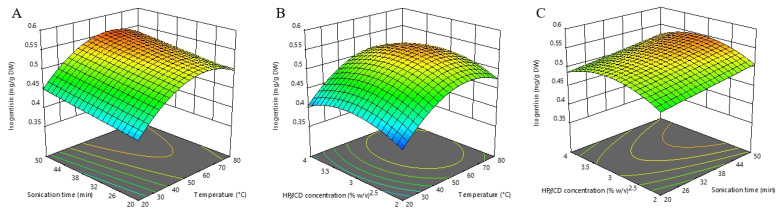
Three-dimensional response surfaces plots of isogentisin as a function of sonication time/temperature (**A**); hydroxypropyl-β-cyclodextrin (HPβCD) concentration/temperature (**B**); and HPβCD concentration/sonication time (**C**).

The pattern of temperature effect on isogentisin extraction (positive linear and negative quadratic term) was similar to that of TPC. As shown in the model equations (Equations (1) and (3)), the influence of the HP*β*CD concentration was also similar to that of TPC in the quadratic term (negative for both), while the linear terms differed (positive for isogentisin and negative for TPC). It seems that adjusting the HP*β*CD concentration can be used as a tool to contribute to selective extraction. The analysis of three-dimensional RSM plots (Figure 3) shows that the peak value is reached at an HP*β*CD concentration of about 3% (*w*/*v*), while its further increase leads to a decrease in the extraction efficiency of isogentisin.

Previous studies show that one of the main strategies to improve the extraction of isogentisin is the use of high concentrations of ethanol in a water-ethanol solvent [26,27,36]. Consequently, the use of HP*β*CD as a co-solvent in a water solvent may be a valuable alternative strategy to improve the extraction of isogentisin if it is necessary to avoid the utilization of organic solvents.

The calculated optimal conditions for maximizing the individual isogentisin response are an extraction temperature of 61.13 °C, an extraction time of 50 min, and a HP*β*CD concentration of 3.11% (*w*/*v*) with the predicted extraction yield of 0.557 mg/g DW.

### 2.3. Multi-Response Optimization and Experimental Validation

For the optimization of the extraction conditions, the developed models and desirability functions were applied. The optimization criteria were “in range” for all independent variables and “maximization” for all response variables simultaneously (Table 3). Weights in response optimization were adjusted to 1 for all targets (neutral setting), and the shape of the desirability function was linear. The calculated global optimal extraction conditions are an extraction temperature of 74.89 °C, an extraction time of 32.57 min, and a HP*β*CD concentration of 3.01% *w*/*v*. The desirability value (D) for this set of optimal extraction conditions was 0.86. Predicted responses under these optimized conditions were 47.90 mg/g DW for gentiopicroside, 0.52 mg/g DW for isogentisin, and 12.86 mg GAE/g DW for TPC. In the final step, experimental validation was carried out by conducting test extraction runs (in three replicates) under the calculated optimal conditions. Experimentally obtained response values of 46.96 ± 1.33 mg/g DW for gentiopicroside, 0.51 ± 0.03 mg/g DW for isogentisin, and 12.99 ± 0.22 mg GAE/g DW for TPC were in good agreement with the predicted values, thus confirming the good predictive accuracy of developed RSM models.

## 3. Materials and Methods

### 3.1. Plant Material

Dried underground parts of willow gentian were obtained from the Production Sector of the Institute for Medicinal Plants Research “Dr. Josif Pančić”, Belgrade, Serbia. Plant material was pulverized by the industrial mill and sieved according to Yugoslav Pharmacopoeia 2000 to obtain a fraction smaller than 0.75 mm, usually treated as a by-product of herbal tea (“dust”). Plant material was stored in paper bags at room temperature during the experiment.

### 3.2. Chemicals and Reagents

Ultrapure water was prepared using a Milli-Q water purification system (Millipore, France). HP*β*CD (97%) was obtained from Acros Organics. Folin–Ciocalteu′s phenol reagent, sodium carbonate, orthophosphoric acid, and methanol were purchased from Sigma–Aldrich Chemie GmbH (Munich, Germany), while HPLC-grade acetonitrile was purchased from Merck (Darmstadt, Germany). Gallic acid (Extrasynthese, Genay, France), gentiopicroside (ChromaDex, Los Angeles, CA, USA), and isogentisin (Phytolab, Vestenbergsgreuth, Germany) were used as standards.

### 3.3. Applied Experimental Design, Modeling, and Optimization

A RSM and Box–Behnken experimental design (BBD) were applied to investigate relationships between extraction conditions as independent variables and the yield of target compounds as dependent variables. The three independent variables (extraction temperature, time, and concentration of HP*β*CD in the water solvent) were varied at three levels coded with −1, 0, and +1 according to the BBD. A total of 17 experimental runs were conducted, including 5 in the central point (Table 1). Experimental ranges for extraction temperature and time were selected considering literature data [26,36], while HP*β*CD concentration levels were chosen according to a preliminary screening study. To describe the relationship between the extraction conditions as independent variables and the extraction yield of gentiopicroside, isogentisin, and TPC as response variables, experimentally obtained values were fitted to the following polynomial model equation:

Y = *β*_0_ + *β*_1_X_1_ + *β*_2_X_2_ + *β*_3_X_3_ + *β*_11_X_1_^2^ + *β*_22_X_2_^2^ + *β*_33_X_3_^2^ + *β*_12_X_1_X_2_ + *β*_13_X_1_X_3_ + *β*_23_X_2_X_3_ (4)

where Y represents yield of target compounds, X_1_-extraction temperature, X_2_-extraction time, X_3_-HP*β*CD concentration, *β*_0_–intercept, *β*_1_, *β*_2_, *β*_3_-linear, *β*_11_, *β*_22_, *β*_33_–quadratic, and *β*_12_, *β*_13_, *β*_23_-interaction regression coefficients, respectively.

Analysis of variance (ANOVA) was employed to assess the influence of independent variables and their interactions on extraction yields of referred compounds. Factors with a *p*-value below 0.05 and those required to support the hierarchy were used to generate predictive models. The adequacy of the generated models was estimated according to the calculated coefficients of determination (R^2^) and *p*-values for lack-of-fit testing and model. For a visual insight into the influence of the independent variables on the response values, three-dimensional RSM plots were constructed. Three-dimensional surface plots were prepared by fixing one of the variables at the central design value and varying the remaining two variables within the studied range.

Optimization of the extraction conditions was carried out according to the desirability function approach. The goal for all independent variables was set to “in range”, while for dependent variables it was “maximization”. The suitability and predictive accuracy of developed models were tested through three trial extractions under predicted optimal conditions. Design of experiment, analysis of obtained data, and optimization of extraction conditions were carried out using Design Expert 11 software (Stat-Ease, Minneapolis, MN, USA)-trial version.

### 3.4. Ultrasound-Assisted Extraction Process

The solid–liquid UAE was performed in ultrasonic water bath (Bandelin Sonorex, Berlin, Germany) with a constant power (320 W) and frequency (35 kHz). Pulverized willow gentian underground parts were extracted with 10 mL of a HP*β*CD water solution at different concentrations (2–4% *w*/*v*) with a constant liquid-to-solid ratio of 30 mL/g. Extractions were conducted at different extraction times (20–50 min) and temperatures (20–80 °C). All experimental runs were performed in Falcon 50 mL conical tubes that were always positioned at the same distance from the transducer. Immediately after the extraction, the solid–liquid mixtures were centrifuged (6000 rpm for 5 min) and filtered through Whatman filter paper No. 1. Samples were stored in glass flasks at 4 °C in a dark place until analysis.

### 3.5. Analytical Methods for Quantification of Target Compounds

#### 3.5.1. HPLC-DAD Analysis

Individual compounds (gentiopicroside and isogentisin) were quantified using the HPLC method described previously by Jovanović et al. (2022) [26]. The instrument Agilent 1200 RR HPLC-DAD (Germany) with reverse-phase analytical column Zorbax SB-C18 (Agilent, Santa Clara, CA, USA), 150 mm × 4.6 mm i.d., 5 μm particle size, was used. The amount of individual compounds was quantified using calibration curves and was expressed in milligrams per gram of dry weight (mg/g DW).

#### 3.5.2. Determination of Total Phenolics Content

The TPC in extracts of willow gentian underground parts were determined using the Folin–Ciocalteu spectrophotometric method described previously by Waterman and Mole (1994) [39]. The extraction yield of TPC was expressed as milligrams of gallic acid equivalents per gram of dry weight (mg GAE/g DW). All measurements were repeated three times.

## 4. Conclusions

In this study, optimization of ultrasound-assisted water extraction coupled with hydroxypropyl-*β*-cyclodextrin (HP*β*CD) as a green co-solvent was successfully applied for the extraction of bioactive compounds from the “dust” of willow gentian underground parts. A response surface methodology (RSM) was performed to investigate relationships between extraction conditions as independent variables (extraction temperature, time, and concentration of HP*β*CD in the water solvent) and the extraction yield of target compounds as response variables (main secoiridoid gentiopicroside, main xanthone isogentisin, and content of total phenolics (TPC)). Extraction temperature was the most influential operating condition, significantly affecting all responses. The calculated optimal conditions providing the highest yield of all responses simultaneously were an extraction temperature of 74.89 °C, an extraction time of 32.57 min, and a HP*β*CD concentration of 3.01% *w*/*v*. The obtained yields in these conditions were in close agreement with the predicted values, suggesting the good predictive accuracy of the developed RSM models.

The reported results contribute to the affirmation of sustainable development through the use of herbal tea by-products and the improvement of knowledge about the novel green extraction techniques needed for the further development of procedures on the industrial scale.

## Figures and Tables

**Table 1:** Box–Behnken experimental design with three independent variables varied on three levels (coded as −1, 0, and 1) and experimentally observed extraction yields of target compounds as dependent variables.

Run	Independent Variables			Dependent Variables		
	X_1_ Extraction Temperature (°C)	X_2_ Extraction Time (min)	X_3_ HP*β*CD Concentration (%, *w*/*v*)	Gentiopicroside (mg/g DW)	Isogentisin (mg/g DW)	TPC (mg GAE/g DW)
1	20 (−1)	20 (−1)	3 (0)	43.56	0.41	10.81
2	80 (1)	20 (−1)	3 (0)	49.03	0.49	13.18
3	20 (−1)	50 (1)	3 (0)	41.51	0.46	12.35
4	80 (1)	50 (1)	3 (0)	48.44	0.51	12.37
5	20 (−1)	35 (0)	2 (−1)	41.76	0.37	11.63
6	80 (1)	35 (0)	2 (−1)	48.85	0.53	12.85
7	20 (−1)	35 (0)	4 (1)	35.74	0.41	10.55
8	80 (1)	35 (0)	4 (1)	48.16	0.47	11.96
9	50 (0)	20 (−1)	2 (−1)	45.19	0.46	12.10
10	50 (0)	50 (1)	2 (−1)	43.87	0.48	12.17
11	50 (0)	20 (−1)	4 (1)	45.54	0.49	12.28
12	50 (0)	50 (1)	4 (1)	43.97	0.54	12.22
13	50 (0)	35 (0)	3 (0)	44.79	0.54	12.26
14	50 (0)	35 (0)	3 (0)	44.64	0.57	12.54
15	50 (0)	35 (0)	3 (0)	42.68	0.52	12.57
16	50 (0)	35 (0)	3 (0)	45.43	0.52	12.49
17	50 (0)	35 (0)	3 (0)	44.85	0.53	12.83

GAE—gallic acid equivalent; DW—dry weight.

**Table 2:** Estimated regression coefficients and analysis of the variance of the fitted second-order polynomial models for the investigated parameters.

	Gentiopicroside	Isogentisin	TPC
	*p* Values		
Linear, quadratic, and interaction terms			
X_1_: Extraction temperature	<0.0001	0.0010	0.0001
X_2_: Extraction time	/	0.0844	0.3910
X_3_: HP*β*CD concentration	/	0.4534	0.0623
X_1_X_2_	/	/	0.0025
X_1_X_3_	/	/	/
X_2_X_3_	/	/	/
X_1_^2^	/	0.0011	0.0190
X_2_^2^	/	/	/
X_3_^2^	/	0.0285	0.0231
Parameters for model assessment			
Model	<0.0001	0.0008	0.0004
Lack of fit	0.1436	0.3397	0.1760
R^2^	0.7533	0.8217	0.8798
Adjusted R^2^	0.7369	0.7407	0.8077
Predicted R^2^	0.6443	0.5267	0.6294

/—negligible significant terms excluded from the model equation.

**Table 3:** Comparison between predicted and experimentally obtained values for investigated responses under calculated optimal conditions.

Response Values (mg/g DW)	Predicted Value	Experimental Value
Gentiopicroside content	47.90	46.96 ± 1.33
Isogentisin content	0.52	0.51 ± 0.03
TPC	12.86	12.99 ± 0.22

DW—dry weight.

## Data Availability

The data that support the findings of this study are available on request from the corresponding author.

## Supplementary Materials

The following supporting information can be downloaded at: https://www.mdpi.com/article/10.3390/molecules27217606/s1, Figure S1: The normal probability plot for gentiopicroside; Figure S2: The normal probability plot for isogentisin; Figure S3: The normal probability plot for total phenolics.

## Sample Availability

Samples of the compounds are not available from the authors.
