# Supplementary material for: Ultrasound-Assisted Water Extraction of Gentiopicroside, Isogentisin, and Polyphenols from Willow Gentian “Dust” Supported by Hydroxypropyl-β-Cyclodextrin as Cage Molecules
Source: Molecules. 2022 Nov 6;27(21):7606. doi: 10.3390/molecules27217606 (PMC9655020; doi:10.3390/molecules27217606)
Supplement: Supplementary file 1 [file molecules-27-07606-s001.zip › molecules-1971317-supplementary.pdf]

molecules

Supplementary Material

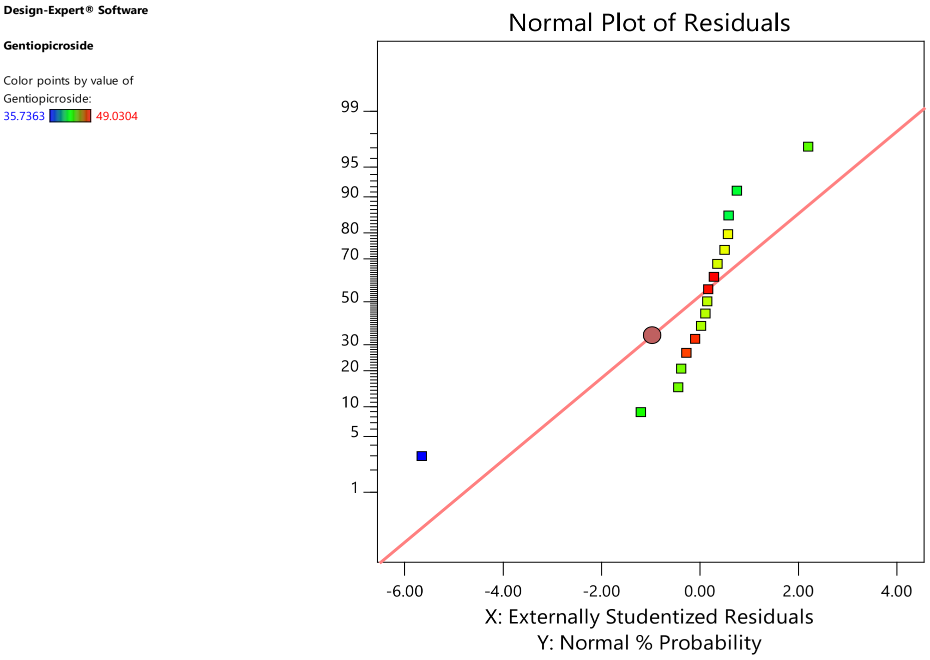

Figure S1. The normal probability plot for gentiopicroside.

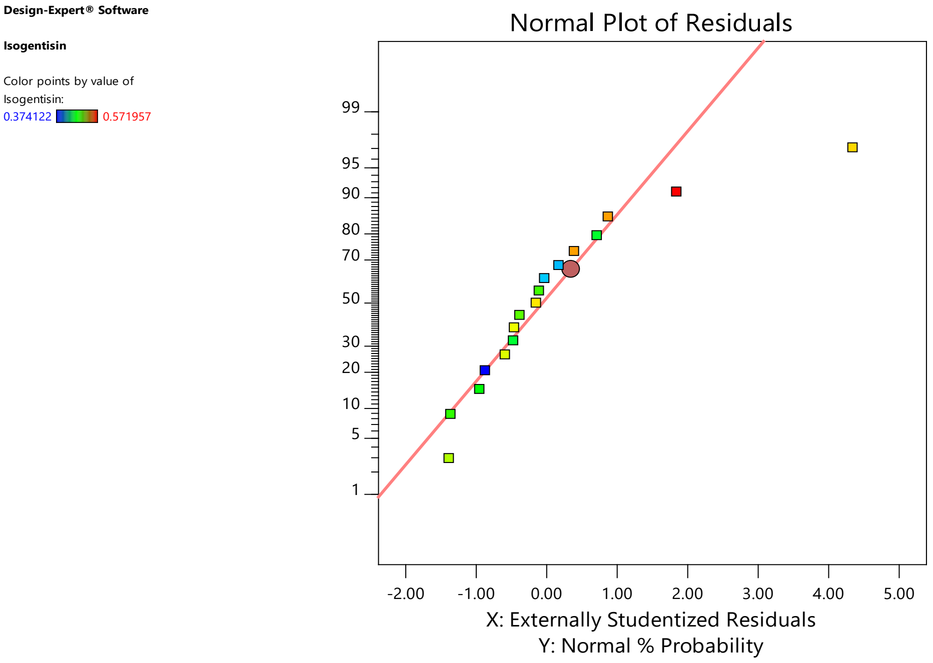

Figure S2. The normal probability plot for isogentisin.

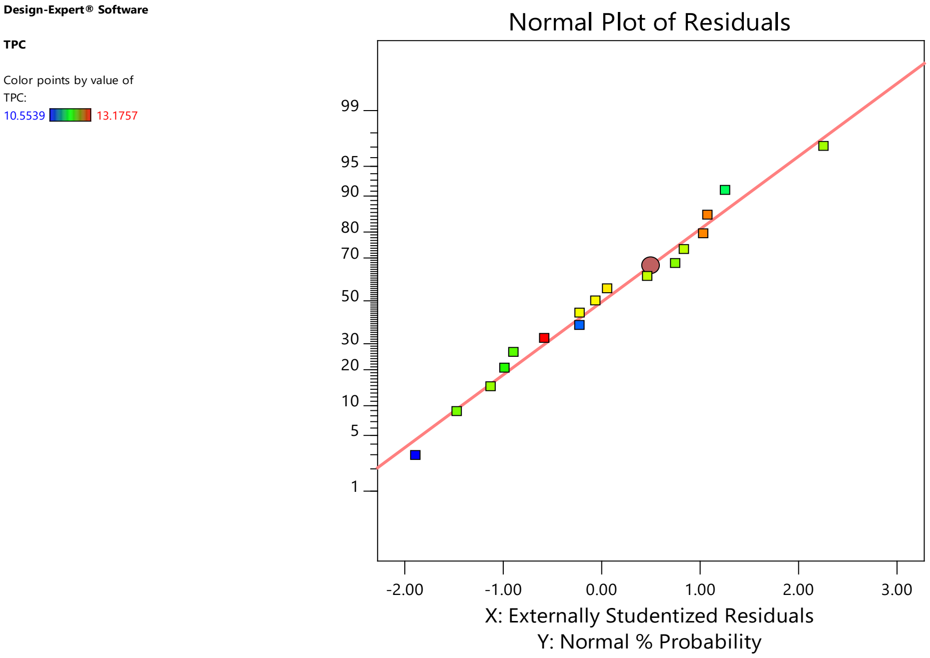

Figure S3. The normal probability plot for total phenolics.
